# Genetic variation in skin traits in New Zealand lambs

**DOI:** 10.1002/jsfa.11844

**Published:** 2022-03-09

**Authors:** Kathryn M McRae, Sue M Cooper, John C McEwan, Rayna Anderson, Wendy E Bain, Hayley J Baird, Ken G Dodds, Shannon M Clarke, Natalie K Pickering, Geoff Holmes

**Affiliations:** ^1^ AgResearch Ltd Invermay Agricultural Centre Mosgiel New Zealand; ^2^ New Zealand Leather and Shoe Research Association (Inc.) Palmerston North New Zealand; ^3^ Focus Genetics Napier New Zealand

**Keywords:** heritability, leather, sheep, GWAS

## Abstract

**BACKGROUND:**

This study explored the genetic variability in the New Zealand sheep population for economically important skin traits. Skins were collected at slaughter from two progeny test flocks, resulting in 725 skins evaluated for grain strain, flatness, crust leather strength and overall suitability for shoe leather. DNA profiles collected from skins post‐slaughter were matched to individual animals using previously collected high‐density genotypes.

**RESULTS:**

Considerable phenotypic variation for skin traits was observed, with around 40% of the skins being identified as suitable for high‐value shoe leather production. Several key traits associated with leather production, including flatness, tear strength, grain strength and grain strain were found to be moderate to highly heritable (*h* ^2^ = 0.28–0.82).

There were no major significant genome‐wide association study (GWAS) peaks associated with many of the traits examined, however, one single‐nucleotide polymorphism (SNP) reached significance for the flatness of the skin over the hindquarters.

**CONCLUSION:**

This research confirms that suitable lamb skins can be bred for use as high‐value shoe leather. While moderately to highly heritable, skin traits in New Zealand lambs appear to be polygenic with no genes of major effect underlaying the traits of interest. Given the complex nature of these traits, the identification and selection of animals with higher‐value skins may be enabled by geomic selection. © 2022 The Authors. *Journal of The Science of Food and Agriculture* published by John Wiley & Sons Ltd on behalf of Society of Chemical Industry.

## INTRODUCTION

The physical properties of livestock skins are important in determining the uses to which the resulting leather can be put. Traditionally, skins have been considered a by‐product of sheep worldwide, aside from breeds such as Karakul and Gotland which have been bred for their pelts.^1^

Between‐breed differences in pelts have been reported, with hair sheep generally having better quality pelts than wool sheep,^2^, ^3^, ^4^, ^5^, ^6^, ^7^ although results can be variable. Estimates of the genetic parameters of ovine skin traits are not common in the literature. Some faults, including ‘ribby’ pelts, pinhole and ‘double‐hiding’,^8^ have been shown to have moderate to high heritability estimates, although the number of records in these studies is often small and the standard errors are high.^9^, ^10^, ^11^ Skin and pelt quality traits in North European sheep breeds have been reviewed,^12^ with traits including skin size, processed weight and thickness all found to have low to high heritability in white Icelandic lambs, ranging from 0.05 to 0.50. This is consistent with heritability estimates in Icelandic sheep,^11^ which were low to moderate for pelt thickness (0.15 ± 0.04) and size of the skin (0.27 ± 0.05), and high for skin weight (0.61 ± 0.06). Heritability estimates of (wool‐on) pelt traits in both Gotland^13^, ^14^, ^15^ and Karakul^16^, ^17^, ^18^ lambs vary between studies, although judgement of pelt quality is largely subjective.

In New Zealand, the national flock is primarily comprised of animals bred and raised for meat production. Post‐slaughter, skins are processed into various leather products and are of relatively low value. If the strength, flatness and uniformity of lamb skins were improved, they could be utilized by the more lucrative and dependable footwear market. Currently, there is only a very limited market for sheep leather in footwear, beyond use in double‐face wool‐on slippers, most of which is supplied out of stronger hair sheep sourced in South Africa.

Previous studies in New Zealand lambs have identified the effects of breed and age at slaughter on leather quality.^19^, ^20^, ^21^ Skin thickness has also been positively correlated with lamb survival in Coopworth lean and fat selection lines,^22^ and preliminary estimates in New Zealand Romney animals indicate skin thickness at 8 months of age has a moderate heritability (0.26 ± 0.22), with a favourable genetic correlation between skin thickness and survival at weaning (0.27 ± 0.04).^23^ There was, however, an unfavourable genetic correlation between skin thickness and fat depth, eye muscle depth, and weaning weight, and the high standard errors associated with all the genetic correlations indicate more data are needed. These studies suggest there may be both genetic and age‐related factors at play in determining the properties of both lamb skin and the subsequent leather produced. This is supported by a preliminary study examining the heritability of skin traits in New Zealand sheep using a subset of data from this study.^24^

The purpose of this study was to investigate whether physical skin properties, identified in the part‐processed and resultant leathers, were under genetic control in New Zealand sheep. If genetic variability exists, this may be exploited to improve the quality of lamb skins and thereby increase their value to farmers and processors.

## MATERIALS AND METHODS

### Animals

Animals were managed following the provisions of the New Zealand Animal Welfare Act 1999, and the New Zealand Codes of Welfare developed under sections 68‐79 of the Act. Skins were collected from progeny from two FarmIQ progeny test flocks, where terminal and dual‐purpose sires (Supporting Information Table S1) were mated to a variety of maternal composite breeds.^25^ Progeny from Flock A were born in 2014, from terminal sire composite rams mated to dual‐purpose (traditionally bred for meat and wool) maternal composite ewes. The ewes were group mated in large groups, with sires assigned to progeny through DNA pedigree. There were 75 sires represented in the data, with an average of four (range 1–14) progeny per sire. Male (*N* = 138) and female (*N* = 149) progeny were slaughtered in February 2015 at a single time point. The average age at slaughter was 173 (± 3) days, with an average carcass weight (CWT) of 19.6 (± 3.3) kg. Progeny from Flock B were born in 2015, from a mixture of dual‐purpose and terminal sires mated to dual‐purpose ewes. There were 24 sires represented in the data, with an average of 18 (range 5–43) progeny per sire. Male (*N* = 331) and female (*N* = 107) progeny were slaughtered in April 2016 at a single time point, with an average age at slaughter of 201 (± 7) days, and an average CWT of 20.2 (± 3.9) kg. CWT records were obtained from the meat processing plant. All other data were obtained from Sheep Improvement Limited (SIL) records.

### High‐density genotyping

Genomic DNA was extracted from ear tissue samples using a high throughput DNA extraction method.^26^ Animals were genotyped using the Illumina Ovine Infinium® HD SNP BeadChip (606 006 markers) according to the manufacturer's protocol. Quality control (QC) checks excluded non‐autosomal markers, those with a call rate below 95%, and/or had a minor allele frequency (MAF) ≤ 0.05. Individuals were excluded from the analysis if there was more than 5% genotyping failure. After QC, 492 463 of the initial 606 006 single‐nucleotide polymorphisms (SNPs) were available for analysis for 715 of the 725 animals for which there were phenotypic measurements.

### Skin processing

Post‐slaughter, all skins were collected and sent to Tomoana Pelt Processors (TPP), Whakatu, New Zealand. The raw skins were fleshed, individually labelled, and a tissue sample was collected for DNA identity matching. The skins were then processed through to the pickle stage at the fellmongery using a standard commercial process. Skins were sprayed with sodium sulphide‐based ‘paint’ and stacked for 3 h before removal of the wool. Skins were then processed in Challenge Cook processors to remove the soluble proteins and open up the fibre structure, delimed and bated to reduce alkaline swelling and further assist with removal of soluble proteins, followed by pickling to stabilize the material before quality assessment and pickle fleshing.

The mechanisms of collagen stabilization in the main tannage process^27^ differed between the two flocks; skins from Flock A were tanned using conventional basic chromium sulphate (BCS), while skins from Flock B were tanned using a novel zirconium sulphate (ZIR) process. Pickled lamb skins from Flock A were processed using BCS at the pilot tannery at the New Zealand Leather & Shoe Research Association (LASRA), in small lots of 50. After the main tanning steps, these small lots were then re‐tanned with mimosa and syntan, dyed brown, then fatliquored. The leather was sammed, hang dried to a moisture content between 16% and 22%, conditioned, stored for 24 h, and staked using a StrojoSvit double‐headed ‘Mollisa’ staker. The leathers were toggled out under tension on the tunnel drier at 40 °C for 30 min for presentation. Pickled lamb skins from Flock B were processed at Lowe Corporation Pacific Leathers, where they were processed in a single lot in a large wooden drum at high speed (10 rpm). The skins were tanned with ZIR and neutralized to a pH similar to those of Flock A. The retanning, fatliquoring and dyeing recipe was identical to the one used on Flock A. The skins were sammed, vacuum dried for 60 s, toggle dried to 20% humidity, seasoned with water, stored for 24 h and then staked using a Baggio synchro staking machine for presentation.

Grain strain was measured on skins from Flock A only, on the depickled skins after treating with Sortassist®, Stahl Holdings BV (Waalwijk, Netherlands), a pH‐dependent blue pigment that highlights grain defects. They were then photographed and scored for the extent and severity of grain strain on the flanks on a scale of 0 (none) to 4 (the most severe/extensive seen). The two flanks of each skin were assessed separately, and the scores added to give a minimum score of 0 and a maximum score of 8. Further testing was conducted on the crusted skins after they were staked and toggled.

Evaluation of flatness was carried out by an experienced assessor, on a scale of 0 (completely flat) to 4 (extreme bumpiness of the surface) on the crusted material. Skins from both flocks were evaluated by the same assessor. Because values vary widely over the skin, with the neck region usually being significantly less flat than the rest of the skin, the neck area, the hindquarters and the rest of the skin were assessed separately. The three figures were then added to give a whole skin grading between 0 and 12.

Samples for physical testing were cut out using press knives on a hydraulic press and conditioned at 20 °C and 65% relative humidity for at least 24 h before testing. Tensile strength and percentage extension of the leather were measured according to International Standard ISO 3376:2002 using an Instron® Model 4467 Universal Testing System. Testing was performed on samples taken in triplicate on each skin, with the results being averaged. From skins of Flock A samples were taken in both parallel and perpendicular relative to the backline, however, in Flock B samples were only taken in parallel.

The tear strength of the leather was measured according to International Standard ISO 3377‐2:2002 using an Instron® Model 4467 Universal Testing System. The results were expressed both as the absolute force required to tear the leather expressed as Newtons (N) and as the force per unit thickness of the sample (N/mm). Testing was performed on samples taken in duplicate on each skin, with the results being averaged. From skins of Flock A samples were taken in parallel and perpendicular relative to the backline, however, in Flock B samples were only taken in perpendicular.

Grain strength and extensibility is an important measure of the resistance to ‘lasting’ during the shoe manufacturing process and were measured in triplicate for each skin using the ball burst test, International Standard ISO 3379:1976. The results for each skin were averaged to give a skin value. Results were expressed as Newtons of force and millimetres of extension. All leathers were also scored on the suitability of the skin as suitable for high‐value shoe leather production (0, no; 1, yes). This was done by the same assessor for both flocks. The suitability score on the leather is a subjective evaluation of the whole skin based on a combination of the looseness assessment after hand stretching the skin in a direction perpendicular to the backbone, gloss on the grain surface, colour levelness, the flatness of grain, ‘softness’ (as measured using a BLC Softness Gauge) and ‘fullness’ of the final leather. Skins are relatively small and to reduce wastage it is important that greater than 80% of the skin is cuttable.

To match the skin number to an individual animal identifier, DNA was extracted from the tissue samples obtained from the fresh skins. A SNP parentage panel^26^ was run on each sample, with the resulting genotypes all matched to stored high density (HD) genotypes from the same animals.

### Statistical analysis

For traits where multiple values were recorded, a trait average was used. Weight and slaughter traits (Tr) were adjusted (aTr) to account for relationships between contemporary group mean and variance, using the equation:

$$\mathrm{aTr}=\left(\mathrm{Tr}/\mathrm{Trm}\right)*\text{oTrm}$$

where Trm is the trait mean for the contemporary group, and oTrm is the overall trait mean for the trait. Parsimonious models for fixed effects and covariates were identified for each skin trait separately via backwards elimination using the generalized linear model (GLM) procedure (SAS Inst. Inc., Cary, NC, USA). Flock, sex, weaning mob (WMOB), and the covariates birthday deviation (BDEV) from the mean of the contemporary group (flock, sex and weaning mob) and CWT were tested; the final fixed effects fitted for each trait are listed in Table 1. Fitting flock in the analysis accounted for differences in the flock, year, and any bias in the processing method.

**Table 1:** Descriptive statistics, heritability estimates and significant fixed effects for skin traits of New Zealand sheep

Trait	*N* ^a^	Mean ± SD^b^	Range	Heritability	*σ*_p_^2^	Fixed effects^d^
Flatness (neck)	723	2.4 ± 0.9	0–4	0.28 ± 0.09 **	0.7	flock CWT
Flatness (hindquarters)	723	1.8 ± 1	0–4	0.39 ± 0.10 **	0.9	flock sex bdev CWT
Flatness (belly)	723	1.6 ± 0.8	0–4	0.43 ± 0.11 **	0.7	flock CWT
Flatness (overall)	723	5.7 ± 2.4	0–12	0.46 ± 0.10 **	5.0	flock sex bdev CWT
Tear strength parallel (N/mm)	282	47.7 ± 6.7	33.8–78.3	0.29 ± 0.19	39.4	sex WMOB CWT
Tear strength perpendicular (N/mm)	710	51.5 ± 9.1	28.1–81.2	0.44 ± 0.09 **	48.7	flock
Tensile extension parallel (mm)	713	36.8 ± 6.1	23.1–92.3	0.11 ± 0.08	35.0	flock sex bdev WMOB
Tensile extension perpendicular (mm)	286	57.6 ± 11	32.4–90.4	NE^c^	NE^c^	CWT
Tensile strength parallel (N/mm^2^)	713	16.71 ± 2.83	10.12–26.06	0.55 ± 0.10 **	6.6	flock sex bdev CWT
Tensile strength perpendicular (N/mm^2^)	285	11.03 ± 1.74	6.23–19.64	0.32 ± 0.19	3.0	sex
Grain strength (Kg)	711	30.7 ± 7.7	12–59.2	0.51 ± 0.10 **	34.4	flock bdev CWT
Grain extensibility (mm)	711	9.3 ± 0.6	6.2–11.3	0.19 ± 0.09 *	0.3	flock sex CWT
Grain strain	288	3.4 ± 1.8	0–8	0.82 ± 0.20 **	3.2	sex WMOB
Suitability (yes = 1/no = 0)	704	0.4 ± 0.5	0–1	0.49 ± 0.11 **	0.2	flock sex bdev CWT

^a^ *N*: number of observations – note a subset of traits were only measured in animals from Flock A.

^b^ SD: standard deviation.

^c^ NE: not estimable.

^d^ All mixed models included animal as a random effect.

Abbreviations: bdev, birth date deviation; WMOB, weaning weight mob; CWT, carcass weight.

*P < 0.05, **P < 0.01

The final dataset used in the analysis contained phenotypic records for 725 lambs (469 male and 256 female; Table 1). Variance components were estimated using restricted maximum likelihood (REML) procedures fitting an animal model in ASReml.^28^ Heritabilities were estimated through univariate analyses of the respective traits, fitting the genomic relationship matrix (GRM) estimated via GenABEL^29^ using the HD genotypes from the 715 animals that passed sample QC.

### Genome‐wide association studies

**Figure 1:**
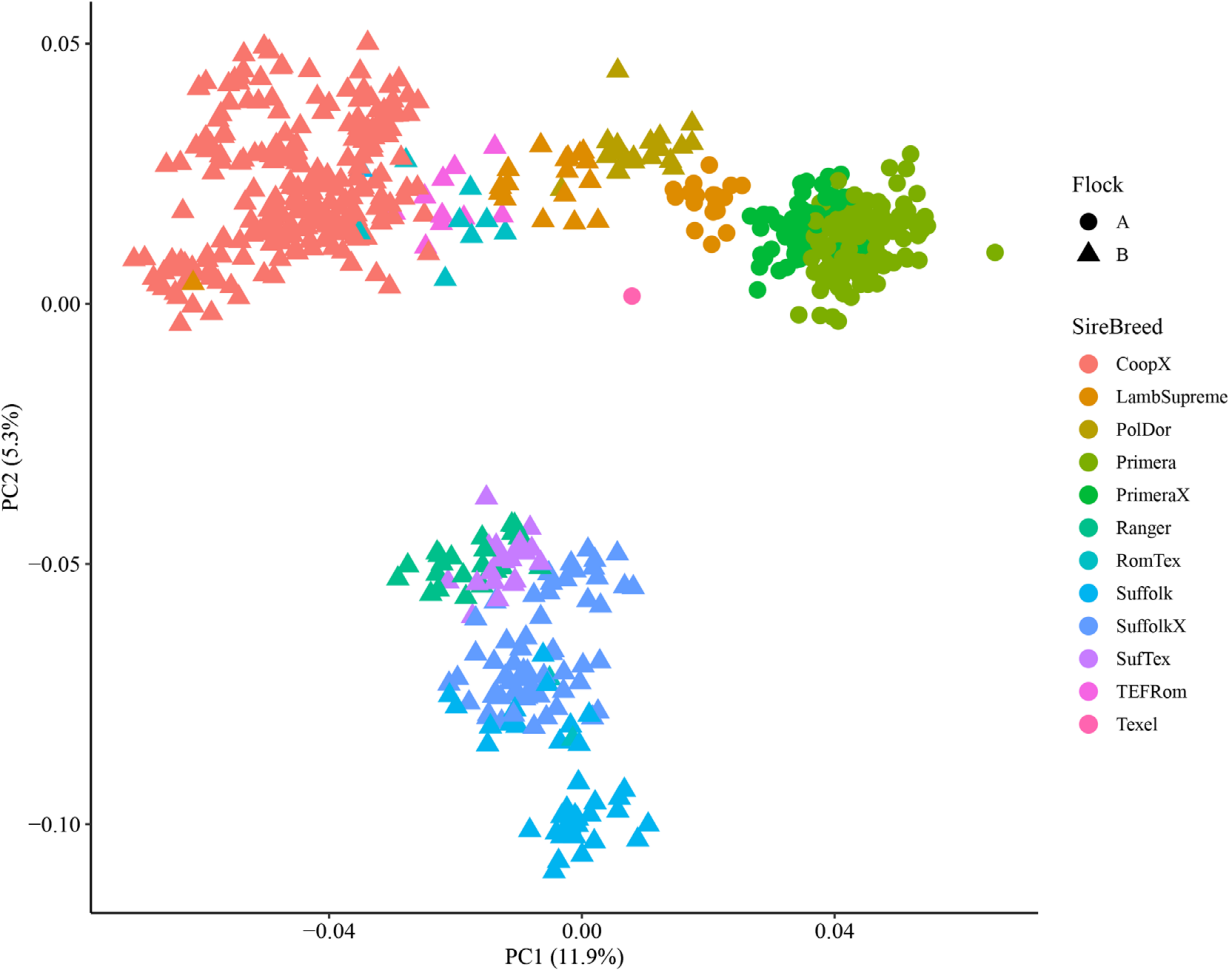
Principal component analysis (PCA) of animals with skin phenotypes. Principal components (PC1, PC2) were calculated from genotypic data using the EIGENSTRAT technique. Markers denote individual animals, with shape representing flock and colour representing sire breed.

Genome‐wide association studies (GWASs) were performed using SNP & Variation Suite version 8.4.0 (Golden Helix, Inc., Bozeman, MT, USA, www.goldenhelix.com) using Efficient Mixed‐Model Association eXpedited (EMMAX) fitting a kinship matrix. Analyses were performed using the covariates reported in Table 1. To account for population stratification not accounted for by flock (Fig. 1) the first two principal components, calculated from genotypic data using the EIGENSTRAT technique,^30^ were fitted as additional covariates. After Bonferonni correction, thresholds were 1.02 × 10^−7^ and 2.03 × 10^−6^ for genome‐wide significance (*P* < 0.05) and suggestive significance (*P* < 0.1), respectively.

## RESULTS

Considerable phenotypic variation was observed for all skin traits. Approximately 40% of skins were classed as being suitable for high‐value shoe leather production (Table 1). In general female skins had lower strength values than males, including tear strength in the parallel (−4.03 ± 0.74) and tensile strength in the perpendicular (−6.27 ± 1.99), but there was very little difference between sexes in overall suitability (0.06 ± 0.04; Table 2). Within a flock, heavier animals, as indicated by CWT, had stronger skins as indicated by grain strength, but again there was little difference in overall suitability. There were several differences between the two flocks which differed primarily in breed composition (Fig. 1), but also to some degree in CWT and age at slaughter. While animals in Flock B were slightly older, overall, the skins were rougher and had lower strength and overall suitability. There were also differences between flocks in the tanning process, with the pickled pelts from flock B processed in a single drum load at a commercial premise. Drum speeds were not as controlled, so processing did introduce more mechanical action than the first flock, which were processed in smaller, more controlled lots at LASRA. The effects of this bias in processing would, however, have been accounted for by fitting flock in the analysis.

**Table 2:** Estimates and standard errors of significant fixed effects for skin traits of New Zealand sheep

	Differences in least squares means		Regression slope	
Trait	Flock (B *versus* A)	Sex (female *versus* male)	bdev^a^ (Δ/d)	CWT^b^ (Δ/kg)
Flatness (neck)	0.68 ± 0.15			0.08 ± 0.01
Flatness (hindquarters)	0.48 ± 0.19	−0.46 ± 0.08	−0.01 ± 0.01	0.05 ± 0.01
Flatness (belly)	0.22 ± 0.16			0.03 ± 0.01
Flatness (overall)	1.41 ± 0.46	−0.84 ± 0.20	0 ± 0.02	0.16 ± 0.03
Tear strength parallel (N/mm)		−4.03 ± 0.74		0.33 ± 0.16
Tear strength perpendicular (N/mm)	−9.52 ± 3.38			
Tensile extension parallel (%)	−3.25 ± 0.81	1.12 ± 0.54	−0.10 ± 0.05	
Tensile extension perpendicular (%)				0.95 ± 0.27
Tensile strength parallel (N/mm^2^)	−20.11 ± 5.8	−11.76 ± 2.25	−0.48 ± 0.25	1.14 ± 0.38
Tensile strength perpendicular (N/mm^2^)		−6.27 ± 1.99		
Grain strength (kg)	−9.74 ± 1.30		−0.09 ± 0.06	1.11 ± 0.09
Grain extensibility (mm)	−0.55 ± 0.08	−0.17 ± 0.05		0.07 ± 0.01
Grain strain		−0.46 ± 0.19		
Suitability (yes/no)	−0.23 ± 0.10	0.06 ± 0.04	−0.01 ± 0	0.01 ± 0.01

^a^ bdev, birth date deviation.

^b^ CWT, carcass weight.

Most traits, where a full dataset was available, had moderate to high heritabilities (Table 1). The heritability of flatness traits ranged from 0.28 ± 0.09 for over the neck to 0.46 ± 0.10 overall. Grain extensibility (0.19 ± 0.09), strength (0.51 ± 0.10) and strain (0.82 ± 0.20) were also moderately to highly heritable. Heritability estimates of traits measured on the smaller subset of data all had larger standard errors and were non‐significant aside from grain strain.

**Figure 2:**
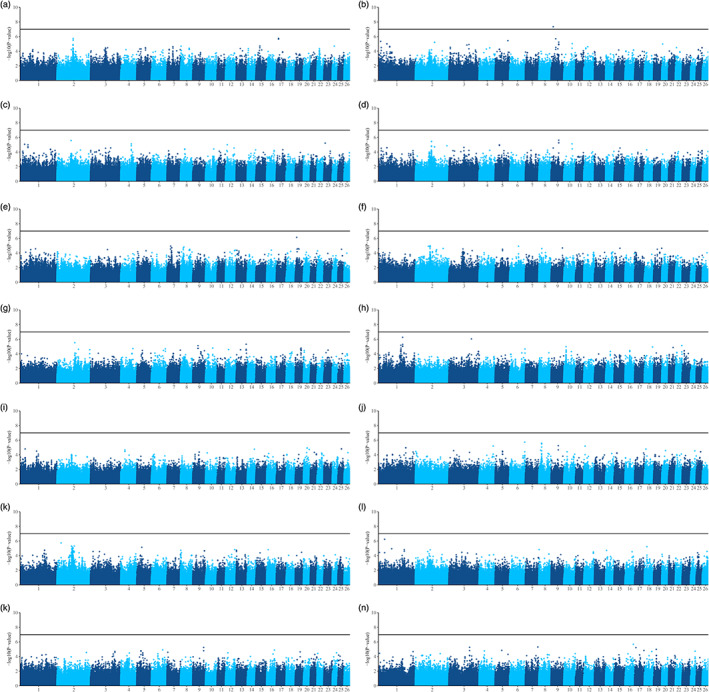
Manhattan plots of genome‐wide association studies for skin traits of New Zealand sheep. Efficient Mixed‐Model Association eXpedited (EMMAX) analysis using a kinship matrix were performed using the covariates reported in Table 1. After Bonferonni correction, the threshold was 1.02 × 10^−7^ (log‐transformed = 6.7) for genome‐wide significance (*P* < 0.05). Individual traits were as follows: neck flatness (A), hindquarters flatness (B), belly flatness (C), overall flatness (D), parallel tear strength (E), perpendicular tear strength (F), parallel tensile extension (G), parallel tensile strength (H), perpendicular tensile extension (I), perpendicular tensile strength (J), grain strength (K), grain extensibility (L), grain strain (M) and overall suitability (N).

Of the 14 skin traits examined (Fig. 2), there was one SNP, rs426878879, that reached significance (*P* = 4.48 × 10^−8^) for the flatness of the skin over the hindquarters. Examination of the QQ plots (Supporting Information Fig. S1) suggested that adjustment for the first two principal components removed any breed composition effects.

## DISCUSSION

Here we report heritability estimates for skin traits estimated using progeny from a mixture of dual‐purpose and terminal sired lambs. These estimates are relatively consistent with previously published estimates using a subset of the data.^24^ This suggests that significant genetic variation in skin traits exists in the New Zealand sheep population and that most traits, including suitability for shoe leather, would be responsive to selection for improvement within a range of breeds.

The suitability of leather for shoe production is a subjective evaluation of the whole skin based on a combination of the traits evaluated in this study. Further work is needed to determine the underlying genetic correlations between the traits reported in this study, along with their relationship with key production traits. While it is unlikely that farmers will breed sheep solely for their skin properties, future work to identify traits of benefit to the farmer, such as lamb survival, that may have a positive correlation with skin strength traits, would give additional reasons to actively improve these properties.

The marker on chromosome 9 that was significant for the flatness of the skin over the hindquarters (rs426878879) is an intergenic variant, located 279.6 Kb from *PTP4A3*, and 413.5 Kb from *ADGRB1*. Other high‐density SNPs in this region were not significant, however, and this region has not previously been identified as a locus associated with traits important to sheep production.^31^ Although not significant, there was a peak observed on chromosome two for several traits, primarily neck and overall flatness, and grain strength. While this region (117–120.120 Mb) includes two collagen genes, *COL5A2* and *COL3A1*, it is also the location of the growth and differentiation factor 8 (*GDF8*) gene. Variation in *GDF8*, also known as myostatin, has been associated with muscling in sheep,^32^ including the Texel breed in New Zealand.^33^ The observed peak may therefore be a result of selection for this mutation by terminal sire breeders.

Combined, the GWAS results suggest that, as expected, multiple genes underly the observable phenotypic differences in lamb skins. Given the complex polygenic nature of these traits, identification and selection of animals with higher‐value skins may be enabled by genomic selection, where markers across the genome are used to predict the genetic merit of an individual.^34^

## CONCLUSION

There is genetic variability in the New Zealand sheep population for several economically important skin traits. Heritabilities of most of the traits measured ranged from moderate to high, including flatness and overall suitability for shoe leather. This indicates that suitable lamb skins can be bred and identified for use as high‐value shoe leather, and if significant premiums existed, relatively fast progress could be made to improve skin quality. Further research is justified to fully define the relationships between skin and production traits and investigate how high‐quality skins can be routinely produced and identified.

## Supporting information


**Figure S1.** Quantile‐quantile (QQ) plots for analyses of skin traits of New Zealand sheep. Observed *versus* expected −log_10_(*P*‐value) for each SNP is plotted. Individual traits were as follows: neck flatness (A), hindquarters flatness (B), belly flatness (C), overall flatness (D), parallel tear strength (E), perpendicular tear strength (F), parallel tensile extension (G), parallel tensile strength (H), perpendicular tensile extension (I), perpendicular tensile strength (J), grain strength (K), grain extensibility (L), grain strain (M) and overall suitability (N).

Figure S2.

**Table S1.** Number of sires and total progeny per breed for each flock.
