# Supplementary figures and images for: Genetic variation in skin traits in New Zealand lambs
Source: J Sci Food Agric. 2022 Mar 9;102(11):4813–9. doi: 10.1002/jsfa.11844 (PMC9546359; doi:10.1002/jsfa.11844)

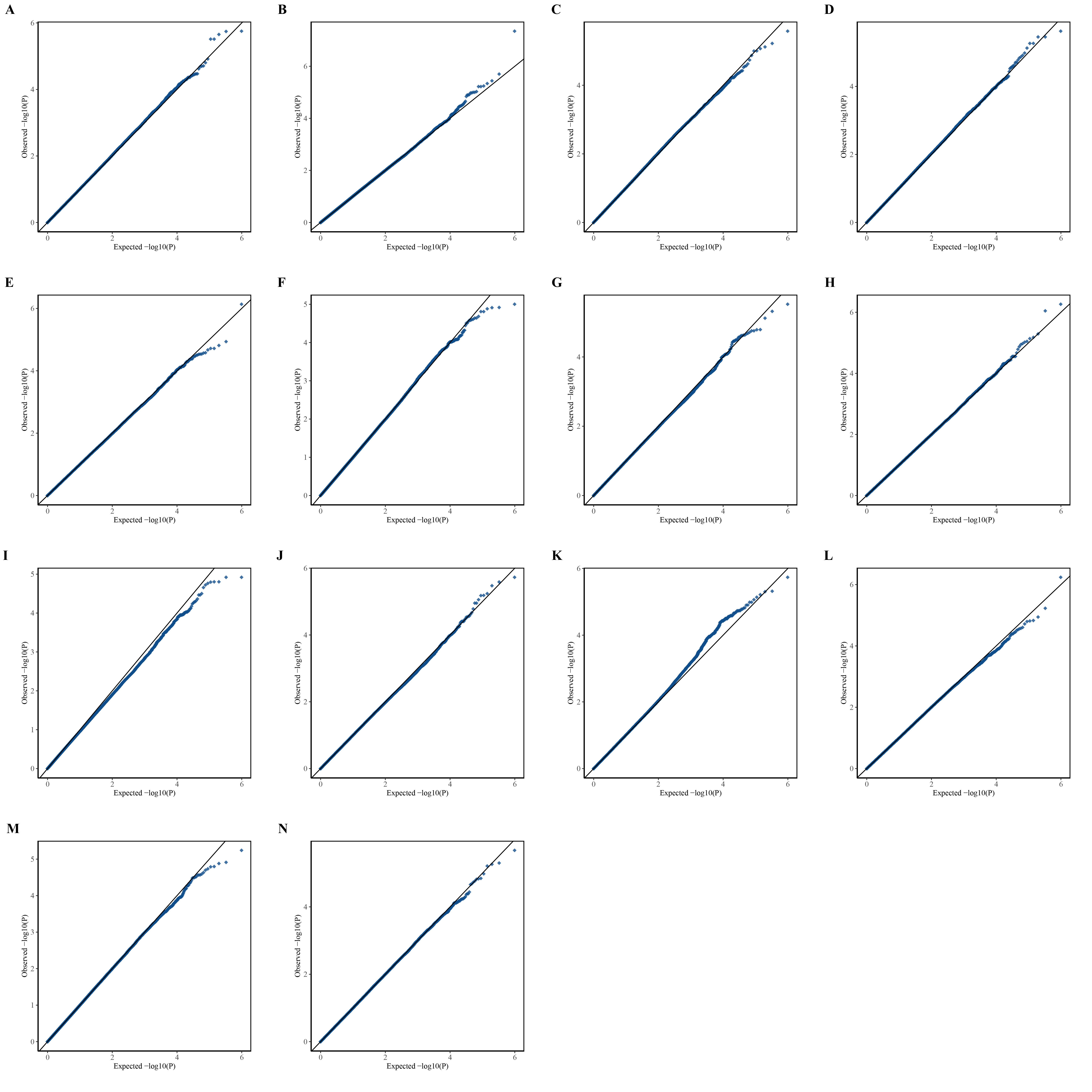

Supplement: Supplementary file 1 — Figure S1. Quantile‐quantile (QQ) plots for analyses of skin traits of New Zealand sheep. Observed versus expected −log10(P‐value) for each SNP is plotted. Individual traits were as follows: neck flatness (A), hindquarters flatness (B), belly flatness (C), overall flatness (D), parallel tear strength (E), perpendicular tear strength (F), parallel tensile extension (G), parallel tensile strength (H), perpendicular tensile extension (I), perpendicular tensile strength (J), grain strength (K), grain extensibility (L), grain strain (M) and overall suitability (N). [file JSFA-102-4813-s002.png]
