# Supplementary material for: Genetic variation in skin traits in New Zealand lambs
Source: J Sci Food Agric. 2022 Mar 9;102(11):4813–9. doi: 10.1002/jsfa.11844 (PMC9546359; doi:10.1002/jsfa.11844)
Supplement: Supplementary file 3 — Table S1. Number of sires and total progeny per breed for each flock. [file JSFA-102-4813-s001.docx]

**Supplementary Table 1.** Number of sires and total progeny per breed for each flock.

|  | **Flock A** | | **Flock B** | |
| --- | --- | --- | --- | --- |
| **Sire breed** | **Sires** | **Total progeny** | **Sires** | **Total progeny** |
| Coopworth cross |  |  | 13 | 228 |
| Lamb Supreme | 3 | 20 | 1 | 18 |
| Poll Dorset |  |  | 1 | 21 |
| Primera | 51 | 198 |  |  |
| Primera cross | 20 | 84 |  |  |
| Ranger |  |  | 1 | 23 |
| RomTex |  |  | 2 | 11 |
| Suffolk |  |  | 1 | 43 |
| Suffolk cross |  |  | 3 | 63 |
| SufTex |  |  | 1 | 21 |
| TEFRom |  |  | 1 | 13 |
| Texel | 1 | 1 |  |  |
| Total | 75 | 303 | 24 | 441 |
